# A rare clinical image of frontal hemorrhagic metastasis in chronic myelogenous leukaemia patient

**DOI:** 10.11604/pamj.2025.50.3.45836

**Published:** 2025-01-02

**Authors:** Pradhyum Dilip Kolhe, Sharath Hullumani

**Affiliations:** 1 Department of Paediatric Physiotherapy, Ravi Nair Physiotherapy College, Datta Meghe Institute of Higher Education and Research (DU) Sawangi Meghe, Maharashtra, India

**Keywords:** Frontal hemorrhagic metastasis, neurosurgery, oncology

## Image in medicine

A 41-year-old male patient was brought to hospital casualty by his relatives with complaints of altered sensorium and reduced consciousness. He had a history of one episode of convulsions, involuntary movements of bilateral upper limb and lower limb. No history of bowel and bladder complaints. He had a known case of chronic myelogenous leukaemia for which he took chemotherapy over the past 4 years. Patient was intubated in casualty. Diagnostic evaluations including a Magnetic Resonance Imaging (MRI) scan, have substantiated a diagnosis of hemorrhagic metastasis in the right frontal region. The figure revealed the presence well defined heterogeneously enhancing T2 hyperintense lesion with edema seen in right precentral gyrus measuring 13x11 mm in size metastases. A well-defined area of blooming appearing hypointense on T2 is seen in right frontal lobe known as acute hematoma metastases. Acute hematoma is seen in right basal ganglia measuring 12x8 mm in size with possible extension into bilateral, 3^rd^ and 4^th^ ventricle. Moderate dilatation of bilateral 3^rd^ and 4^th^ ventricle noted with periventricular chronic fatigue syndrome (CSF) leak. Mass effect is seen in form of midline shift of 8 mm towards left side. Patchy areas of diffusion restriction are seen in parasaggital cortex of frontoparietal lobe and body of corpus callosum likely acute infarct in bilateral anterior cerebral artery (ACA).

**Figure 1:**
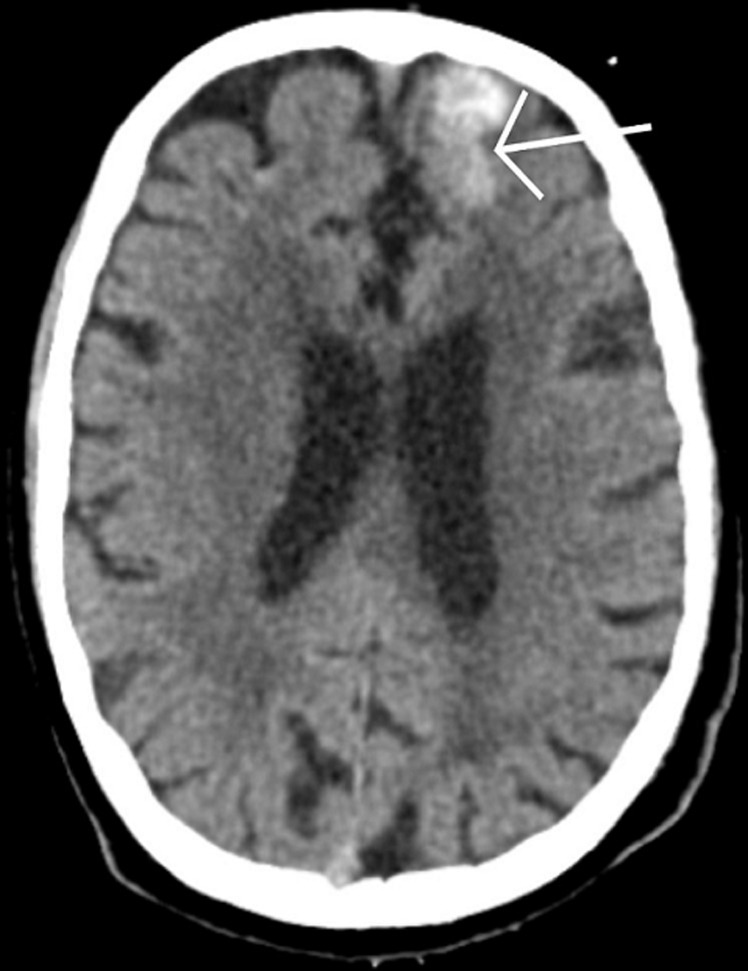
acute hematoma metastases in right frontal lobe

